# A Trip to South Africa

**Published:** 1902-04

**Authors:** J. Massie

**Affiliations:** Veterinary Major, Royal Canadian Field Artillery, Kingston, Canada


### A TRIP TO SOUTH AFRICA.

By J. Massie, Veterinary Major,
ROYAL CANADIAN FIELD ARTILLERY, KINGSTON, CANADA.

In January, 1900, a brigade division of artillery, consisting of "C," "D," and "E" batteries, Royal Canadian Field Artillery, six breech-loading twelve pounders each, under command of Col. Drury, proceeded to South Africa on active service.

I had the honor of accompanying this part of my regiment in my capacity as veterinary officer.

We proceeded to Halifax by rail, and leaving that port on board S. S. Laurentian January 21st, we arrived at Cape Town, South Africa, February 18th, being twenty-eight days en route.

It must be considered that in leaving our country in mid-winter for a sea voyage of some six thousand miles, and crossing the equator with a shipload of horses in the hold and on mid-decks, and men in every quarter of the ship, that the constitutional strain on men and horses was most severe.

To make matters worse, or as bad as possible, before embarking the horses they were exposed to a very heavy, cold rain. In fact, many of the animals had already taken cold during transit by train, the weather during the previous few days being extremely cold.

There was no help for it, the animals had to be put on board cold and wet, and were put in temporary stalls constructed of posts and scantling, and fitted with slings.

For three days after leaving Halifax we experienced heavy weather, and nearly every horse on board developed lung trouble in some form.

To say that the duties of a veterinary officer and his staff for twenty-eight days and nights under the circumstances were trying in the extreme would be putting it very mildly indeed.

We lost twenty-six animals during the trip, nearly all from pneumonia.

On arriving at Cape Town we disembarked our horses in a rather weakened condition; many had to be helped down the gangways, but I was thankful to get them ashore once more, as I was sure they would only need a few weeks' rest and care with good feeding to get them all right again.

Such was not our good fortune, however, as we were scarcely two weeks in camp at Green Point (just outside the town) when we were ordered up country with all speed by train from Cape Town, we knew not where.

After travelling nearly two days and a night, we were detrained at a place called Victoria, and on the following day formed part of a column with mounted infantry marching through what is known as the Karoo desert, five hundred miles, where not a blade of grass was to be seen, nothing but a small shrub growing from six to eight inches high, resembling Scotch heather, on which sheep and goats can live, but which our horses would not touch, and where water was scarce and of poor quality. Many of our horses died from exhaustion on this march, which was the hardest I experienced in the campaign. Forage was scarce, and consisted for a time of three or four quarts daily of wheat and five pounds of wheat chaff per horse. Supply transport wagons, drawn by mules and oxen, carried food and other necessaries; but these supplies were not always replenished when needed, as in a hostile country transport was not safe, and was always slow and difficult.

As a general thing we were issued ten pounds of oats and fourteen pounds of pressed hay per horse per diem, eight pounds of corn (called mealies) for the mules; trek oxen, fifteen pounds of hay and allowed what they could get when safe to graze.

In parts of the Orange River Colony, late Orange Free State and Transvaal, good grazing was found, and in my opinion are among the best stock-raising countries in the world. Animals need very little care, and can feed on the veldt grass all the year round. I have seen as good fat cattle in Africa as any Canadian farmer could wish for.

The diseases most commonly met with in horses are sand colic; the animals being always hungry will keep nibbling at any green root or grass or other plant they can get, eating sand in their grazing, large quantities entering the stomach and bowels.

In autopsies that I have made I have found the lining membrane fairly rubbed off in parts, and the organs generally much inflamed. Ticks also infest horses as sheep are in this country. Sore eyes, glanders, mange, and what is known as *horse sickness.* This disease is not well understood, nor is its cause known.

The disease usually makes its appearance in the latter part of the summer and autumn, and disappears with the first frosts. There are two forms of the disease, one chiefly affects the lungs and in which there is no external swellings. In the other form, known as *dilkop*, swellings occur about the throat, head, and neck. In both forms the mortality is great, especially when epidemic.

Premonitory symptoms are not well marked, especially in hard-worked and poorly-fed animals. Dulness is perhaps noticeable, the animal may not refuse his food, and temperature remains almost or quite normal. He suddenly begins to blow and have difficulty of breathing, a hollow cough, and discharge from the nostrils of a white, frothy character, sometimes tinged with blood. This frothy substance is very abundant, and falls to the ground like soapsuds. The animal becomes unable to move, and stands with the forelegs stretched out and head low. Death usually occurs in from one to three hours. Horses which have recovered from an attack of the disease are said to be *salted*, and are considered immune, and will sell for much more than animals not known to be salted. But cases are known where animals have taken the disease a second time and died.

Pleuropneumonia contagiosa (called lung sick) is often found in cattle; also rinderpest, which carried off thousands of animals in Cape Colony a few years ago.

Horses from many different parts of the world were brought into South Africa for military and transport service, and in such enormous numbers as to greatly enhance their prices in the home markets, but I cannot dwell on the respective merits or demerits of the different classes, as I am afraid my article is already too long to be interesting.

No doubt the question of horse-breeding will receive the attention it deserves in South Africa, and the many valuable mares which have found their way into the country from England, Australia, India, New Zealand, the United States of America, and Canada will in time be the progenitors of as fine a type of horse as any country can produce.

The native stock is largely in-bred with Arab blood, and are remarkably good saddle and light harness animals, averaging from fourteen to fifteen hands, and possessing wonderful endurance and remarkable soundness.

I had the honor of being chief veterinary officer on three different columns, having, including trek mules and oxen, from three to five thousand animals. I was furnished with ample supplies of medicines for the outfit; also a cart, mules, and two Kaffir drivers, besides my orderly.

